# How can we recruit more men of African or African-Caribbean ancestry into our research? Co-creating a video to raise awareness of prostate cancer risk and the PROFILE study

**DOI:** 10.1186/s40900-022-00347-9

**Published:** 2022-04-18

**Authors:** Emma Hainsworth, Eva McGrowder, Jana McHugh, Elizabeth Bancroft, Sean Mahabir, Winston Webber, Rosalind Eeles, Susanne Cruickshank

**Affiliations:** 1 Royal Marsden NHS Foundation Trust, 203 Fulham Rd, London, SW3 6JJ UK; 2 The Institute of Cancer Research SRD Building, 1st Floor D1S5, 15 Cotswold Road, Sutton, SM2 5NG Surrey UK; 3 Patient Author, London, UK; 4 Faculty of Health Sciences and Sport, University of Stirling, Stirling, UK

**Keywords:** Patient and public involvement, Inclusion, Diversity, Prostate cancer, Co-creation

## Abstract

**Background:**

Men of African ancestry are at increased risk of developing prostate cancer (PrCa) compared to men from other backgrounds. The PROFILE study aims to understand whether genetic information can better target who needs PrCa screening. PROFILE has so far had difficulty reaching men of African or African -Caribbean ancestry to take part. In this involvement project we worked in partnership with a group of such men to co-create a video to raise awareness of PrCa risk amongst this community and promote participation in the study.

**Methods:**

We recruited seven men of African or African-Caribbean ancestry who completed an initial survey on the Cancer Patients’ Voice platform. We then held an online discussion panel and maintained contact to encourage dialogue and planning of the video. Utilising a participatory approach, the ideas for the video were decided in collaboration with the panel who held expert knowledge of various communities and understood the messages that would best resonate and engage with other men of the same origins. Once the video had been edited and finalised, two members of the group expressed interest in writing up the project and are listed as co-authors.

**Results:**

The video in its entirety was driven by the panel’s ideas. The choice of a barber shop setting; leading with a positive case study and highlighting the importance of men’s family members rather than a focus on scientific language, statistics or researchers were all features that were discussed and agreed upon by the panel. The men shared the video within their networks. It was placed on websites and promoted as part of a social media campaign during Black History Month.

**Conclusions:**

Groups with the greater healthcare needs and the most to gain from advances in care and treatment can often be the most excluded from research participation. This is pertinent in PrCa research where men of African or African-Caribbean ancestry are at greater risk. The project gave equal power and decision making to the men and provides an example of successful inclusive involvement. The result was a unique approach to making a study video.

## Plain English summary

This project involved working in partnership with men of African or African-Caribbean ancestry to co-create a video intended to raise awareness of prostate cancer (PrCa) risk and promote participation in a genetic screening study called PROFILE. Men of African or African-Caribbean ancestry are at increased risk of developing PrCa compared to other men. The PROFILE study aims to understand whether genetic information can better target who needs PrCa screening. The study has had problems recruiting men from these communities. Methods: We engaged seven men of African or African-Caribbean ancestry: three PROFILE study participants and four from the Race, Ethnicity and Cultural Heritage (REACH) staff forums across the Royal Marsden Hospital and the Institute of Cancer Research. They completed a survey, joined an online discussion panel and we continued working together. The group decided on the structure and content of the video; to include a PrCa survivor who had been successfully screened and treated early for his disease, and a daughter of one of the panellists. The men were also involved in the dissemination plans of the finished video, and two agreed to be co-authors of this paper. Findings: Features of the video led by the men included the choice of a barber shop setting; leading with a positive shared story and highlighting the importance of family rather than science, statistics or researchers. Dissemination: The group shared the video within their networks. It was placed on websites and promoted as part of a social media campaign during Black History Month.

## Background

This paper reports a patient and public involvement project conducted in partnership with men of African or African-Caribbean ancestry to plan, create and disseminate a video to raise awareness of prostate cancer risk and promote participation in a genetic prostate cancer screening study, the PROFILE study (NCT02543905). For clarity in terminology, we refer herein to men of African or African-Caribbean ancestry as men of black African or black African-Caribbean ancestry—which is defined within the study protocol as having both parents and all four grandparents of that origin.

### The PROFILE study

Prostate cancer is the most common cancer in men in the United Kingdom (UK) with men of African or African-Caribbean ancestry at 2–3 times the risk of developing it in comparison to their counterparts of European ancestry, and with a 30% higher mortality rate [1]. We do not understand enough about why some men, including men of African or African-Caribbean ancestry and men of European ancestry with a family history of the disease are at higher risk. The PROFILE study aims to address this gap in the knowledge by looking at the genes of healthy men at higher risk including men of African or African-Caribbean ancestry. The PROFILE study monitors for signs of prostate cancer using blood tests, and if required, scans and biopsies are undertaken, and participants are followed up over 5 years. The aim of the research is to understand whether genetic information can be used as a screening tool to target those at higher risk of developing prostate cancer. To date, the PROFILE study has experienced some difficulty in recruiting men of African or African-Caribbean ancestry and we wanted our involvement work to address this. Evidence in the literature supports using involvement activity to improve study recruitment, particularly if this involvement includes working in partnership with people who have relevant lived experience [2, 3].

## Inclusive involvement

In this project we wanted to work in partnership with a group of men of African or African-Caribbean ancestry and share learning so that we could create a video that would best engage these communities. In doing so and writing up our experience we hope to add to the limited evidence that exists on inclusive involvement.

We know that the groups with the greater healthcare needs and the most to gain can be the most excluded from healthcare decision-making. Covid-19 has made everyone more aware of health inequalities and has starkly highlighted the unacceptable situation of poorer health outcomes for patients from Asian and Black backgrounds [4]. There is evidence that these groups are less likely to have participated in research [5] and that more widely, the UK health system often mirrors the forces that undermine the health of people from ethnic minority backgrounds [6]. There are numerous barriers to ethnic minority participation in research which are complex, and these include structural issues ranging from lack of access to care, distrust in healthcare systems, to discrimination. It is therefore crucial to report and describe involvement which explicitly attempts to address power imbalances and reach groups that have been underserved in such a way so that evidence can be built, and lessons can be shared to improve health equity.

There is a current drive encouraged by major national funders to offer public involvement opportunities that are accessible and reach people and groups according to research need. Despite being an ambition, it is not clear that this is happening to any great extent. There is a considerable gap in the literature with only a very small number of health and social care studies outside of the United States (US) including any African or African-Caribbean and other minority ethnic involvement. Those studies that have written about them, showed that involvement was limited to the early stages of the research process, with poor reporting on the details of facilitators or barriers [7].

### Power relations

Public involvement in research means research that is done ‘with’ or ‘by’ the public, not 'to', 'about' or 'for' them [8]. Despite this aspiration of a more equal partnership, current models of Patient and Public Involvement (PPI) can often reproduce imbalances of power within healthcare where patients/public can find themselves invited into spaces which give them little scope to do anything other than legitimise decisions which have been made by others [9, 10]. This can be seen in professionally dominated approaches with a business meeting or committee format which maintains the appearance of inclusion but has the effect of excluding those who may not feel comfortable in this setting. We wanted to conduct our involvement activity with a more equal power balance between researchers and those involved.

### Measuring impact

The increased focus on measuring the impact of involvement activity can have the effect of applying scientific, rigid notions of the methods of measurement to a process which can be more a social collaboration with learning between researchers and the public as a valuable end in itself [11]. The impact of involvement is not limited to the experience of the patient or public participants and the effect on the research project, researchers themselves also learn new knowledge through involvement which can change their priorities and attitudes [12, 13]. Our project aimed to offer an inclusive involvement opportunity that would follow the approach of participatory research by encouraging the development of equal power relationships and shared learning. In our reporting we have adopted a qualitative narrative rather than the use of a standardised checklist as we feel that this better reflects the social collaborative partnership of the work [14]. We ensured that involvement continued throughout the project by including two men from the group as co-authors in our publication.

## Methods

### Aims and objectives

Our objective was to work in partnership with a group of men of African or African-Caribbean ancestry to plan and co-create a video. This video had two aims: to raise awareness about prostate cancer risk in men from these communities, and to encourage participation in an existing genetic screening study, PROFILE.

### Recruitment into discussion panel

We held a discussion panel of seven men of African or African-Caribbean ancestry in an online meeting via ZOOM™ Video Communication in December 2020. The panelists came from a mix of African and African-Caribbean backgrounds and a range of ages. Three were existing PROFILE study participants who had been approached by members of the clinical team prior to speaking to the authors; four others were members of what was then known as the Black, Asian and Minority Ethnic (BAME) staff forums, now called the Race, Ethnicity and Cultural Heritage forums (REACH). One further member of this forum was not able to attend the online meeting. His work role meant that he did not have access to a computer or the necessary free time to attend the discussion. In this instance one of the authors met him in person during one of his breaks. All members of the group were reimbursed for their time according to the National Institute for Health Research (NIHR) National Standards for Public Involvement [15]. In the discussion they shared their ideas about what representation and messages they thought should be included in the proposed video to engage men within the African or African-Caribbean communities.

### Participation in the video

In the discussion panel, men agreed that the video should feature a ‘success story’ of someone who had been diagnosed early with prostate cancer due to screening. We used our clinical network to help approach a man whose prostate cancer had been detected early and successfully treated to share his story within the video. It was also felt to be important to include a loved one or family member to share their perspective on the importance of their relative being aware of prostate cancer risk and screening, so we also included a daughter and her father, who was taking part in the PROFILE study. We included two more PROFILE study participants from our discussion panelists, and this made a total of five participants who we agreed were to be filmed within the video. The group was given the opportunity to review and comment upon the draft edit before it was finalised.

### Writing up the project for publication

We wanted the collaborative nature of this project to extend beyond the making of the video and into the dissemination stage. Some of the group members were active within their own community networks and were encouraged to share the finished video across these networks. The opportunity to co-author this paper was advertised to all who were involved in this involvement project, and two members expressed their interest to take part in the writing process. We held an online meeting lasting one hour, where they agreed to be co-authors, and in which they discussed priorities for what should be included. We then communicated by email in the exchange of draft text for comment and approval. The focus of their contribution was their views on the important learning from the project and how the video had been shaped by the involvement of the group. They were also happy to be included as named authors.

## Results

### How was the video shaped by the group’s input and ideas?

#### Leading with a positive case study

The group agreed that positive stories would be the most impactful and there was widespread support for starting the video with a case study of a man who had been screened early, treated for prostate cancer, and was now living well. They thought that this would provide a challenge to commonly held beliefs within communities that cancer was ‘a death sentence’ and could help to dispel fears. The video ended with the same man stating: *‘You can survive cancer; I have survived cancer.’*

#### The importance of family members

At the planning stage the group thought it would be important to include family members’ perspectives on men looking after their health as this would resonate with their own relationships and would provide personal motivation. Talking about their personal prostate cancer risk or seeking help for symptoms could then be seen within a context of being strong and caring for your loved ones and making sure that they do not experience worry. As the prostate is linked to a masculine identity and virility, it was thought important to frame the topic in a way which did not threaten this identity. The group discussed the way in which women play their part in keeping healthy for their family by having breast and cervical screening; therefore, men being aware of prostate cancer risk and seeking help could be seen as men doing the same, and ‘playing their part’. This is another example of the positive and proactive framing that the group believed would best engage the audience. Reflecting on the finished video, two of our patient/public authors agreed that the daughter encouraging her father to become aware of prostate cancer risk and to be checked out for any symptoms in the same way that women do routinely, conveyed a powerful message:

‘Support from family, from wives, partners, children, I think that’s really good to egg men on to get the checks done’ (SM, public co-author).

WW thought that this message might also inspire female viewers to speak to their male relatives:

‘A wife could watch it and think of her husband having symptoms, or a daughter might think of her father, and they might now feel confident to say something’ (WW, patient co-author).

#### Prostate cancer affects younger black men too

Men who appeared in the video were of different ages to reflect the inclusion criteria of the PROFILE study (40 to 69 years). The inclusion of younger men in the video was designed to challenge a commonly held perception that prostate cancer or cancer in general is only a problem for an older age group. WW felt that their participation in the study role-modelled proactive health behaviour which was not common amongst his own contemporaries:

‘Here are men who have not yet been diagnosed, willing to be monitored, they’re willingly and joyfully adhering to it…..That’s a message that so far I’ve found difficult to pass on to my friends’ (WW, patient co-author).

SM’s participation in the project as a member of the planning group and a co-author had an influence on his own behaviour, so much so that he requested a PSA test as part of a routine health-check as a result; something he would otherwise not have thought to request.

#### No scientific language, statistics, or researchers

The men unanimously agreed that we should avoid scientific language, statistics, death rates and tragedies as this would immediately cause people to switch off. They thought that researchers or the clinical team should not play a prominent role in the video and that since the messaging was aimed at healthy individuals, that the setting should be neutral and away from a clinical background. This was a surprise to the researchers and challenged their preconceived ideas about what the video might look like and stood in striking contrast to many of the cancer awareness and study promotion videos in circulation, which included just that sort of clinical setting and style of information.

#### The barber shop and its emotional resonance

On camera, men spoke with warmth and feeling about the importance of a barber shop in their lives. The location of the video had been unanimously agreed by the group as a setting which represented a social meeting place, where sensitive subjects could be discussed safely and freely, and where men got away from the pressures of everyday life and felt better about themselves. Talking about something as personal and sensitive as prostate cancer risk felt easier in this venue:

‘You are just free to talk…it allowed us to be so natural. We’re not doing a clinical interview; it’s just a barber shop’ (WW, patient co-author).

### The video’s impact on recruitment into the PROFILE study

The PROFILE study recruits men for targeted prostate cancer screening in two cohorts: men of African or African- Caribbean Ancestry and men of European Ancestry with a family history of prostate cancer. Initially, the PROFILE study opened in 2009 as a pilot study (Integrated Research Application System (IRAS): 7951; Research Ethics Committee (REC) reference: 09/H0801/19) with just the European ancestry family history cohort. In 2015, the PROFILE full study opened with two cohorts: men of African or African- Caribbean Ancestry and men of European Ancestry with a family history of prostate cancer (IRAS: 132999; REC: 13/LO/1787; NCT02543905). Table 1 shows recruitment figures in both cohorts since the study began in 2015, with the family history cohort recruitment remaining relatively constant over the years. However, recruitment to the African or African-Caribbean cohort was poor, with no recruitment at all to this cohort during the first year of this study (2015) when compared to 47 participants being recruited to the family history cohort in the same year. The first participant to the African or African-Caribbean cohort was recruited in 2016, and this being the only recruit to this cohort until 2018, when compared to 96 participants recruited to the family history cohort during the period of 2016–2018 (Table 1). From 2018, initiatives were taken by the study team to improve recruitment to the African or African-Caribbean cohort, including the introduction of the study to Primary Care recruitment with multiple GP practices; study posters; engagement efforts within this community and recently, the PPI engagement video to further raise awareness of prostate cancer risk in this population group. The video was made available online and was promoted from August 2021 alongside a wider initiative with a mail-out conducted by GP practices highlighting the health risk to black men. The recruitment figures in the African or African-Caribbean cohort have increased three-fold in 2021 compared to previous years. Although it is not possible to attribute all of this increase to the video, this trend in recruitment is encouraging and does suggest a positive impact.

**Table 1:** PROFILE recruitment figures

Recruitment year	Men of African and African-Caribbean ancestry recruited	Men of European ancestry with Family history of prostate cancer recruited
2015	0	47
2016	1	69
2017	0	27
2018	10	53
2019	32	47
2021	103	45

## Discussion

This was an involvement activity which set out to centre the voices of men of African or African-Caribbean ancestry and facilitate the delivery of their ideas for raising awareness and engagement around prostate cancer risk. The PROFILE study had previously tried several approaches to raise awareness in the black community without significant success. Some of these included engaging community leaders such as church leaders, counsellors, community volunteers, general practitioners; displaying study posters in local businesses, churches, leisure centres; attending community meetings and holding awareness talks. All these approaches were well received by the public; however, they did not fully translate in men expressing interest in the PROFILE study or to seek further information about prostate cancer. What was striking from these public talks was that it was mainly women who were keen to seek more information about prostate cancer, the types of symptoms to look out for and generally, what the PROFILE study was about. They would take study material, participant information sheets to pass on to their male family members, with the hope that they would then contact the study team and or take part in the study. Very few men expressed interest in the study through these approaches. The issue was not the lack of identifying participants, but rather failure to engage them fully. We therefore had to change our strategy and focus on raising awareness in these communities.

A recent NIHR public contributors’ feedback survey of 2019 revealed that there was a lack of diversity in the people who took part in involvement activity, with younger people and minority ethnic groups under-represented [16]. This gap is clear within the literature. Describing this work of inclusive involvement from the conception of the project through to its dissemination is an important addition. By writing up this activity descriptively, it is hoped that this will usefully contribute to existing evidence which can inform and guide future inclusive projects.

Cultural views and beliefs can affect the way men of African or African-Caribbean ancestry engage with information about cancer risk and screening, and health information based solely on the clinical meaning of cancer can be incongruent [17]. By seeking the group’s views on what features of a video would resonate culturally with men of African or African-Caribbean ancestry, we hoped to engage these communities more effectively. The barber shop setting was an example of this, presenting the researchers with a novel idea that challenged their preconceptions and led them to venture outside of the comfort zone of the biomedical setting to find somewhere in the community that fitted the brief. The setting was remarked upon as one of the most engaging aspects of the video and testament to the need for researchers to be led in directions that did not feel familiar or immediately comfortable.

This project was funded by an NIHR Biomedical Research Centre (BRC) award dedicated to support a discrete Patient Public Involvement and Engagement activity. As such, it could accommodate the time required to build and sustain relationships; time which is not always available or well-aligned to the more usual research funding timetables [18]. As the planning work was held at a time of Covid-19 restrictions, the discussions had to take place online. In some senses this made for more equitable participation as it did not require travel or attendance in person. In the case of one member of the group whose work did not accommodate the use of a computer, one of the authors met him separately in his place of work. This prioritisation of relationships over the usual structure of formal meetings worked well to address power differentials which can stifle involvement [19] and in this case facilitated contributions.

### Limitations

There is a large range of cultural and ethnic diversity within the African and African-Caribbean population in the UK and the seven men who participated in this project, although from a variety of backgrounds, could not represent this diversity in its entirety. These men were members of the staff REACH forums, study participants and a cancer survivor, and as such, were likely to be positive about medicine and research. Although they had an understanding of the issues, they did not represent the voices of men living in these communities who may have feelings of disenfranchisement and mistrust of the healthcare system in the UK.

## Conclusions

This project provides an example of a successful inclusive involvement activity which has been written up descriptively to share good practice and learning for future work trying to increase diversity within study recruitment. The video created by the involvement group contained features and messages designed to resonate culturally with men of African or African-Caribbean ancestry and move beyond the provision of purely clinical information. Both the final video and the process of its creation aimed to engage and build trust to ensure that the study could better recruit and benefit those men most affected by prostate cancer.

## Data Availability

The full length video can be accessed via YouTube™ using the following link: https://www.youtube.com/watch?v=A72hCjLN3gE

## Abbreviations

BAME: Black, Asian and Minority Ethnic
BRC: Biomedical Research Centre
IRAS: Integrated Research Application System
NHS: National Health Service
NIHR: National Institute for Health Research
PPI: Patient and Public Involvement
PrCa: Prostate Cancer
PSA: Prostate-Specific Antigen
REACH: Race, Ethnicity and Cultural Heritage
REC: Research Ethics Committee
UK: United Kingdom
US: United States
